# Prevalence and co-occurrence of parentally reported possible asthma and allergic manifestations in pre-school children

**DOI:** 10.1186/1471-2458-13-764

**Published:** 2013-08-16

**Authors:** Kristina Bröms, Dan Norbäck, Margaretha Eriksson, Claes Sundelin, Kurt Svärdsudd

**Affiliations:** 1 Department of Public Health and Caring Sciences, Family Medicine and Preventive Medicine, Uppsala University, Uppsala, Sweden; 2 Centre for Clinical Research Uppsala University/County Council of Gävleborg, Gävle, Sweden; 3 Department of Medical Sciences, Occupational and Environmental Medicine, Uppsala University, Uppsala, Sweden; 4 Department of Women’s and Children’s Health, Paediatrics, Uppsala University Hospital, Uppsala, Sweden

## Abstract

**Background:**

The aim of this study was to make an in-depth analysis of the prevalence and co-occurrence in pre-school children of possible asthma and atopic manifestations.

**Methods:**

In Sweden 74%-84% of preschool children, depending on age, attend municipality organised day-care centres. Parents of 5,886 children 1–6 years of age, sampled from day-care centres in 62 municipalities all over Sweden, responded to a postal questionnaire regarding symptoms indicating prevalent possible asthma, allergic rhinitis, eczema, and food, furred pet and pollen allergy and other data in their children. Possible asthma was defined as any of the four criteria wheezing four times or more during the last year, physician diagnosis and current wheezing, ever had asthma and current wheezing, and current use of inhalation steroids, all based on questionnaire responses.

**Results:**

The overall prevalence of possible asthma was 8.9%, of eczema 21.7%, of rhinitis 8.1%, and of food allergy 6.6%. There was a highly significant co-occurrence between possible asthma and all atopic manifestations, 35.7% having any of the manifestations. Presence of pet allergy was the manifestation showing the closest co-occurrence with presence of possible asthma, presence of pollen allergy with presence of rhinitis, and presence of food allergy with presence of eczema. Assessed from plots of age-specific prevalence of possible asthma, rhinitis, eczema and food allergy, the prevalence of all manifestations increased from one to three years of age and then decreased, except for rhinitis where the prevalence increased until six years of age, indicating no specific ordered sequence.

**Conclusions:**

Parentally reported possible asthma, eczema and food allergy had a curvilinear prevalence course across age with a maximum at age 3, while rhinitis prevalence increased consistently with age. Co-occurrence between possible asthma and atopic manifestations was common, and some combinations were more common than others, but there was no evidence of a specific ordered onset sequence.

## Background

In recent decades, the prevalence of allergies and asthma in industrialized countries has increased, particularly among children, although the increase rate may have levelled off during the last few years [1,2]. According to the International Study of Asthma and Allergies in Childhood (ISAAC), the prevalence of asthma and rhino-conjunctivitis among 6- to 7-year-old children in Sweden in 2002 was in the middle of the international range, whereas the prevalence of eczema was the highest of all countries participating [1,3]. Food allergy prevalence has been less investigated, and the ranking of nations is therefore less certain [4-6].

There are few published studies on age-specific prevalence of asthma, allergic rhinitis henceforth called rhinitis, eczema, and food allergy [7,8]. Most studies have been based on certain age groups, for instance 4-year olds, or 6- to 7-year olds, etc., which makes comparisons between studies difficult [3,9]. It is well known that that there is co-occurrence between asthma and various atopic manifestations. However, the information on the extent of co-occurrence is still fragmentary [9-11].

What is known as the ‘atopic march’ hypothesis [12-14] is closely linked to this co-occurrence. According to the hypothesis, the onset of atopic manifestations tends to come in an ordered sequence, for instance eczema tends to be followed by asthma. The ideal design to test the hypothesis would be a large, longitudinal cohort study with frequent re-examinations. However, the hypothesis could also, at least provisionally, be tested with a cross-sectional study design, given that the onset of the various atopic manifestations always follows the same pattern.

Most Swedish pre-school children attend a day-care centre (DC) [15]. Such centres may therefore be suitable sampling frames for studies of these age groups. The aim of this cross-sectional study was to make an in-depth analysis of the co-occurrence in pre-school children of possible asthma, allergic rhinitis, eczema, and food allergy.

## Methods

### Setting

Sweden is one of the most sparsely populated countries in Europe, the median population density being 26 people per square kilometre, and 80% of municipalities have 82 people or fewer per square kilometre [16]. The corresponding numbers for the municipalities included in this study were 57 and 130 people. For administrative purposes, Sweden at the time of the data collection was divided into 25 regions and 290 municipalities, the smallest administrative unit.

All Swedish pre-school children are entitled by law to day-care (DC) organised by the local municipality. In 2002, 74% of all pre-school children attended DC, somewhat fewer among the youngest children and more than 80% of children of 3 or older [15]. The vast majority of DCs are run by the local municipal administration. The few privately operated DCs are all subcontracted to the municipal administration and follow the same set of rules as publicly operated DCs. A DC may have one to four sections. At the time of the study 15–20 children were cared for in each section. Many sections had children of all ages, but some were age stratified (1–3 years or 4–6 years). The day-care fees are heavily subsidised by the municipalities, parents usually paying about 10% of the real cost. A detailed description of choice of day care has been given elsewhere [17].

During the 1990s special DCs for children with asthma or allergies, ‘allergen avoidance day-care centres’ (AADCs) were established at the initiative of municipal school administrations, parents, local politicians, and local DC staffs. The operations, set of rules, and fees are the same as for ordinary day-care centres (ODC) with the exception that AADCs give priority to children with asthma or allergies, but accept other children as well, space permitting. As shown in a previous report all AADCs had strict regulations to avoid pet, smoking, perfume, and dust exposure [18]. The ODCs usually had no such regulations.

### Study population

In the late 1990s all 72 AADCs in Sweden were identified as previously described in detail [18]. The two geographically closest ODCs to each AADC were chosen as control centres. Later, a few AADCs were closed and a few new ones were opened, leaving 70 AADCs with 84 sections and 140 ODCs with 440 sections for this study, in 62 municipalities, all over Sweden. One third of the AADCs were located in the same building as a control ODC. The indoor and outdoor environments at these day-care centres have been reported elsewhere [18].

The addresses of the 1,412 children attending the AADCs and the 7,345 children attending the ODCs were obtained from the local school authorities. A questionnaire was mailed to the parents of these children. Responses were obtained regarding 1,001 AADC children (70.9%) and 4,958 ODC children (67.5%), after two reminders when necessary. Of the respondents, 1,000 AADC children and 4,886 ODC children were 6 years old or younger. The ODC children constitute the study population for this report, except in the prevalence calculations, where also data from the AADC children was used.

### Questionnaire

The ISAAC written screening questionnaire with questions about asthma and wheezing, eczema, and rhinitis, extensively used all over the world and regarded as the gold standard for postal questionnaires on childhood asthma, rhinitis, and eczema, was used [1]. Although intended for children 6 years or older, it has been validated down to 3 years old with good results [19]. For this study, supplementary questions on medical treatment, physician assessed asthma, rhinitis and eczema diagnosis, food allergy, allergy to pollen or furred pets, parental education, smoking habits, and some additional variables not reported here, were added (see Additional file 1).

Regarding possible asthma, the following criteria were used: 1) ever had asthma and has current symptoms (wheezing in the last 12 months), or 2) wheezing 4 times or more during the last 12 months, or 3) physician diagnosis and current symptoms, or 4) current use of inhalation steroids. Regarding rhinitis, the following criteria were used: 1) ever had hay-fever and has current symptoms (sneezing or a runny or a blocked nose when the child did not have a cold or the flu), or 2) rhino-conjunctivitis without cold last 12 months, or 3) physician diagnosis and current symptoms, or 4) being on anti-allergy medication and having current symptoms.

The following eczema criteria were used: 1) itchy rash coming and going for at least 6 months in typical eczema localisations in the last 12 months, or 2) physician diagno-sis and current symptoms (itchy rash), or 3) current use of steroid ointments and current symptoms. The following food allergy criterion was used: Reported food allergy to milk, egg, fish, peanuts, nuts, soy, or stone fruits, but not including lactose or gluten intolerance.

Parental education was classified as a nine-year compulsory education (=1), a two-year upper secondary education (= 2), a three-four-year upper secondary education (= 3), or university or other tertiary education (= 4).

The study was approved on several occasions before and during the data collection process, first by the Research Ethics Committee at Uppsala University and later by the National Research Ethics Board.

### Statistical analysis

The statistical analyses were conducted using the SAS software, version 9.3 [20]. Partial non-response (missing data in returned questionnaires) was on average 0.6% with a maximum for individual variables of 1.1%. Simple (crude) differences between groups in proportions were tested with the Mantel-Haenszel chi-square test.

**Figure 1:**
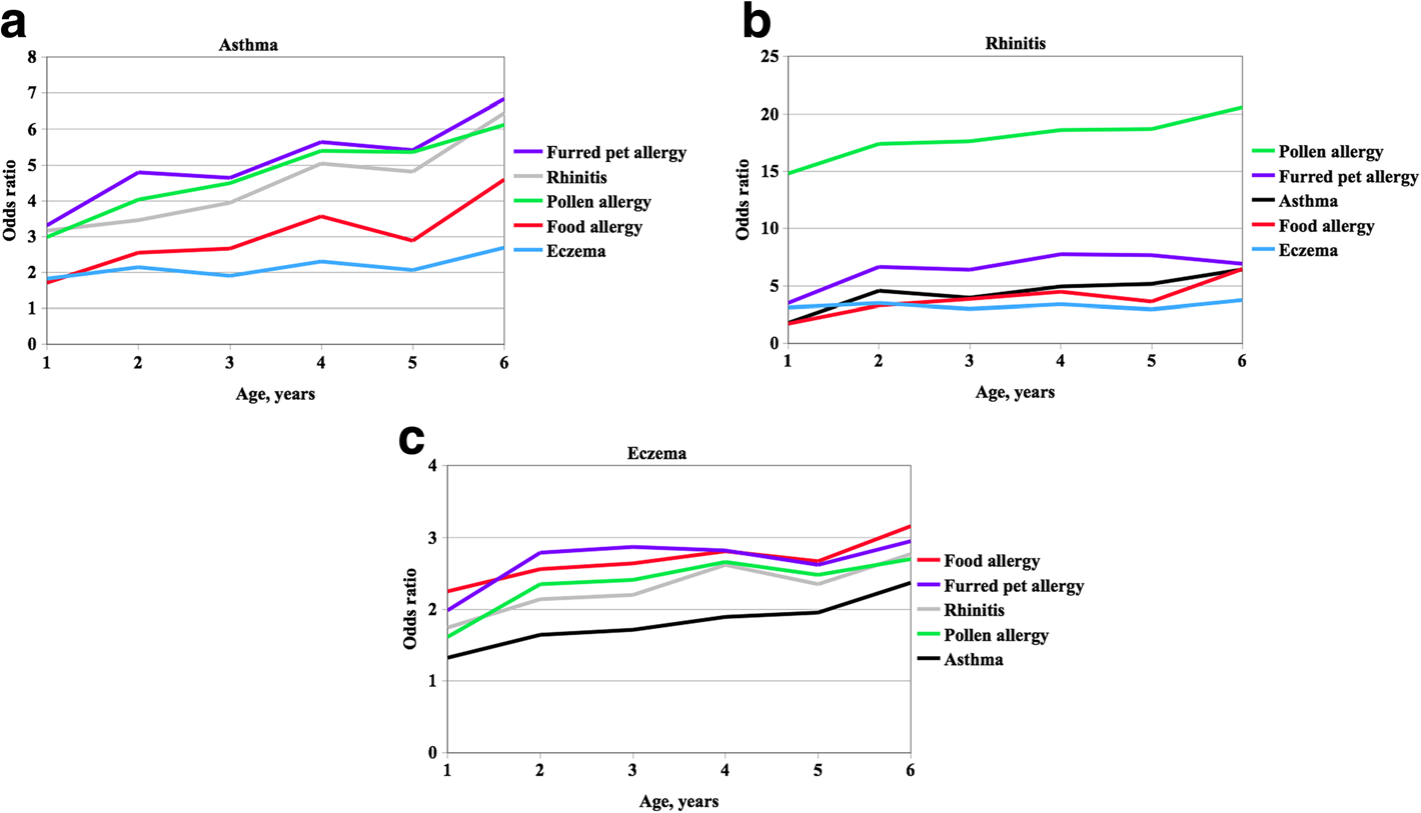
**Odds ratio of co-occurrence.** Odds ratios by age for co-occurrence of possible asthma versus furred pet allergy, rhinitis, pollen allergy, food allergy and eczema **(a)**, rhinitis versus furred pet allergy, possible asthma, pollen allergy, food allergy and eczema **(b)**, and eczema versus furred pet allergy, rhinitis, pollen allergy, food allergy and possible asthma **(c)**.

A possible asthma variable (0 = no, 1 = yes) was created based on fulfilment of any of the criteria presented above. A rhinitis, eczema, food allergy, furred pet and pollen allergy variable was constructed accordingly. The variables selected were those of primary interest for the hypothesis. The analyses of co-occurrence between possible asthma, rhinitis, eczema, food, furred pet and pollen allergies were performed with multiple logistic regression analysis with presence of possible asthma, rhinitis and eczema (yes/no) as dependent variables, one at a time, and the others as independent variables, first by age (Figure 1) and then adjusted for the influence of age and sex (Table 1). The analytical technique provides odds ratios (OR), confidence intervals (95% CI), and Wald’s chi-square. The latter is the test variable on which the p-value is based. Consequently, Wald’s chi-square may be used to rank the impact of the independent variables on outcome.

**Table 1:** Co-occurrence

	**Possible asthma**				**Rhinitis**				**Eczema**			
	**OR**	**95% CI**	**Wald’s chi2**	**p**	**OR**	**95% CI**	**Wald’s chi2**	**p**	**OR**	**95% CI**	**Wald’s chi2**	**p**
Age	0.86	0.80-0.92	16.3	<0.0001	0.97	0.88-1.06	0.5	0.49	1.0	0.95-1.05	0.04	0.85
Sex	1.43	1.16-1.77	11.2	<0.001	1.02	0.78-1.33	0.01	0.90	0.82	0.71-0.95	7.5	<0.01
Possible asthma	___	___	___	___	2.68	1.90-3.79	31.4	<0.0001	1.47	1.17-1.86	10.5	<0.005
Rhinitis	2.71	1.93-3.81	33.2	<0.0001	___	___	___	___	1.94	1.46-2.58	20.5	<0.0001
Eczema	1.48	1.17-1.87	10.9	<0.001	1.99	1.49-2.65	22.1	<0.0001	___	___	___	___
Food allergy	1.76	1.29-2.41	12.6	<0.0005	2.07	1.41-3.05	13.6	<0.0005	3.44	2.72-4.34	107.8	<0.0001
Pet allergy	3.22	2.22-4.66	38.4	<0.0001	1.55	0.96-2.48	3.3	0.07	2.28	1.63-3.19	23.4	<0.0001
Pollen allergy	1.92	1.29-2.85	10.4	<0.005	35.39	25.23-49.64	426.5	<0.0001	1.59	1.12-2.23	6.9	<0.01

Effects on the prevalence of current possible asthma, rhinitis, and eczema in logistic regression analyses based on questionnaire data among boys and girls 1–6 years old, attending ordinary day-care centres.

Most of the municipalities represented in this study had only one AADC and two ODCs sampled, irrespective of population size, resulting in under-representation of large municipalities in the prevalence calculations. To adjust for this circumstance, an analytical model was employed in which the total number of children, by age and sex, in each municipality represented in the study was downloaded from Statistics Sweden [21]. The number of children with possible asthma, rhinitis, eczema and food allergy by age and sex in the municipality was then computed, based on the prevalence of these conditions in the municipality-specific ODC study populations, after which the number with these diseases in the municipality-specific AADC study population was added. National prevalence was obtained by pooling the municipality-specific data on number of cases in relation to exposed population size. The procedure has been described in detail elsewhere [17].

Age was used as a continuous variable and defined as age attained at last birthday. The age-specific prevalence functions were computed with logistic regression technique. The fit between the crude age-specific prevalence and prevalence data generated from the analysis model was tested with a logistic regression technique. To check for non-linearity a model including a second and a third degree age polynomial, and interaction between sex and age were tested. The best fit between observed and expected outcome was obtained with a second degree (possible asthma and rhinitis) or a third degree (eczema and food allergy) age polynomial and an interaction term between sex and age, explaining 50% of the prevalence variation. On scrutiny, the model fit appeared excellent. All tests were two-tailed. The level of significance was set at p < 0.05.

## Results

### Characteristics of the study population

Of the 4,886 ODC children, 2,476 (51%) were boys. The number of 1-year-old children was 203 (4%), 2-year-olds 916 (19%), 3-year-olds 976 (20%), 4-year-olds 1,017 (21%), 5-year-olds 1,168 (24%), and 6-year-olds 606 (12%). The age distribution was similar in the AADC children. Regarding parental education, 268 (5%) children had parents with compulsory education only, 1,032 (21%) had at least one parent with two-year upper secondary education, 1,459 (30%) had at least one parent with three- or four-year upper secondary education, and 2,127 (44%) had at least one parent with college or university education.

### Possible asthma, rhinitis, eczema, and food, furred pet, and pollen allergy

Possible asthma, rhinitis and eczema manifestations are presented by age groups in Table 2. The prevalence of possible asthma manifestations was stable or decreased by age, while the prevalence of rhinitis manifestations was stable or increased, and the prevalence of eczema manifestations was generally stable, except that ointment treatment became less prevalent by age.

**Table 2:** Manifestation prevalence

	**Age groups, years**			
	**1-2**	**3-4**	**5-6**	**p**
n	1119	1993	1774	
Asthma manifestations, n (%)				
Ever had asthma and current^*)^ symptoms	81 (7.3)	153 (7.7)	94 (5.3)	<0.05
Wheezing 4 times or more last 12 months	77 (6.9)	94 (4.7)	64 (3.6)	<0.0001
Physician diagnosis and current^*)^ symptoms	69 (6.2)	142 (7.2)	88 (5.1)	0.10
Being on inhalation steroids	57 (5.1)	129 (6.7)	88 (5.0)	0.64
Any asthma criterion	114 (10.2	195 (9.8)	132 (7.4)	<0.01
Rhinitis manifestations n (%)				
Ever had hay-fever and current^*)^symptoms	43 (3.8)	86 (4.3)	105 (5.9)	<0.01
Rhino-conjunctivitis without cold last 12 months	49 (4.4)	97 (4.9)	94 (5.3)	0.26
Physician rhinitis diagnosis and current^*)^symptoms	10 (0.9)	35 (1.8)	58 (3.3)	<0.0001
On anti-allergy medication and current^*)^ symptoms	17 (1.5)	41 (2.1)	66 (3.7)	<0.0001
Any rhinitis criterion	79 (7.1)	137 (6.9)	145 (8.2)	0.20
Eczema manifestations, n (%)				
Itchy rash ever, coming and going for ≥ 6 months, and itchy rash in typical eczema localisations last 12 months	224 (20.4)	416 (21.0)	383 (21.8)	0.31
Physician eczema diagnosis and current^*)^ symptoms	102 (9.2)	198 (10.0)	183 (10.4)	0.31
Steroid ointments last 12 months and current^*)^ symptoms	98 (8.8)	141 (7.1)	117 (6.6)	<0.05
Any eczema criterion	240 (21.5)	444 (22.3)	398 (22.4)	0.56

Prevalence n (%) of current possible asthma, rhinitis and eczema manifestations based on questionnaire data in boys and girls 1–6 years old attending ordinary day-care centres.

^*)^ Current indicates symptoms of the specific disease at any time during the last 12 months.

The prevalence of food, furred pet and pollen allergies is shown in Table 3. The prevalence of egg allergy decreased by age, while the prevalence of peanut and nut allergy increased. All other food allergies had a stable prevalence. The prevalence of all allergies to furred pets increase by age and so did the pollen allergy prevalence.

**Table 3:** Allergy prevalence

	**Age groups, years**			
	**1-2**	**3-4**	**5-6**	**p**
Reported food allergy				
Milk allergy	38 (3.4)	88 (4.4)	75 (4.3)	0.34
Egg allergy	37 (3.3)	48 (2.4)	33 (1.9)	<0.05
Fish allergy	11 (1.0)	15 (0.8)	19 (1.1)	0.70
Peanut allergy	13 (1.2)	37 (1.9)	42 (2.4)	<0.05
Nut allergy	14 (1.3)	28 (1.4)	41 (2.3)	<0.05
Soy allergy	6 (0.5)	16 (0.8)	8 (0.5)	0.63
Stone fruit allergy	9 (0.8)	20 (1.0)	24 (1.4)	0.15
Any food allergy	78 (7.0)	150 (7.5)	140 (7.9)	0.34
Allergy to furred pets				
Cat allergy	21 (1.9)	55 (2.8)	77 (4.4)	<0.0001
Dog allergy	9 (0.8)	45 (2.3)	57 (3.2)	<0.0001
Horse allergy	0	19 (1.0)	34 (1.9)	<0.0001
Rodent allergy	4 (0.4)	24 (1.2)	41 (2.3)	<0.0001
Any allergy to furred pets	25 (2.3)	79 (4.0)	103 (5.9)	<0.0001
Pollen allergy	42 (3.8)	94 (4.8)	124 (7.1)	<0.0001

Prevalence of current food, furred pet and pollen allergy based on questionnaire data in boys and girls 1–6 years old attending ordinary day-care centres.

### Age specific prevalence

**Figure 2:**
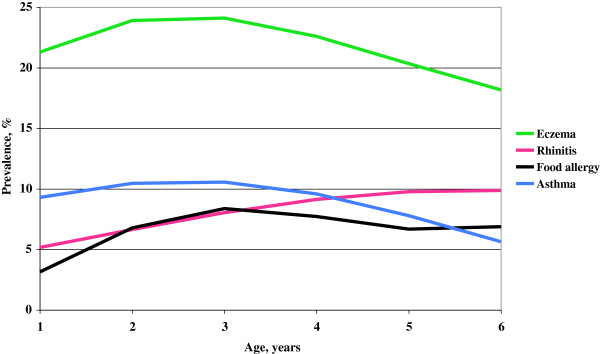
**Overlap between parentally reported possible asthma, rhinitis, eczema and food allergy.** Age specific prevalence (%) of possible asthma, rhinitis, eczema, and food allergy among Swedish pre-school children based on application of study questionnaire data to Swedish national data on preschool children.

The age-specific prevalence of possible asthma, rhinitis, eczema and food allergy adjusted for sampling municipality size is presented in Figure 2. The mean prevalence across age was 21.7% for eczema, 8.9% for possible asthma, 8.1% for rhinitis, and 6.6% for food allergy. The prevalence of the most common condition, eczema, increased during the early years of life from 21.3% at age 1 to 24.1% at age 3 and then decreased to 18.2% at age 6. The possible asthma prevalence increased from 9.3% at age 1 to 10.6% at age 3 and then decreased to 5.7% at age 6. The rhinitis prevalence increased from 5.2% at age 1 to 9.9% at age 6. Food allergy prevalence increased from 3.2% at age 1 to 8.4% at age 3, and was then fairly stable.

### Co-occurrence of possible asthma, rhinitis, eczema and allergies

**Figure 3:**
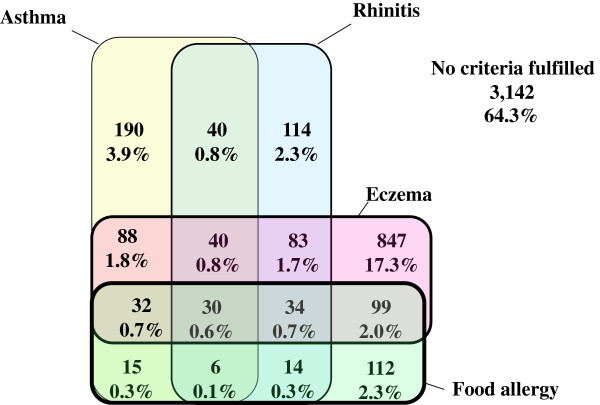
**Parentally reported prevalence.** Venn diagram showing overlap between presence (%) of possible asthma, rhinitis, eczema and food allergy in children 1–6 years old attending ordinary day-care centres. Percentages refer to crude number of children with criterion or criteria combinations in relation to total ordinary day-care study population.

Crude co-occurrence among the ODC children is illustrated in the Venn diagram in Figure 3, showing that 35.7% had any of the manifestations. The most common manifestations or manifestation combinations were eczema only, possible asthma, rhinitis, food allergy, and various combinations of eczema, possible asthma and rhinitis, and food allergy.

Statistical measures of co-occurrence between prevalent possible asthma, rhinitis, eczema, and allergies to food, furred pets and pollen in multiple logistic regression analyses are displayed by age in Figure 1a-c and summarised across age in Table 1. Presence of possible asthma (dependent variable) was highly significantly related to the presence of rhinitis, eczema and all the allergy variables (independent variables), after adjustment for the influence on possible asthma prevalence of age and sex (co-variates), indicating a relationship between the presence of possible asthma and presence of all the other variables. The variables with the largest impact on possible asthma prevalence, as indicated by Wald’s chi-square, were in ranked order furred pet allergy, rhinitis, food allergy, eczema and pollen allergy. As shown in Figure 1a the co-occurrence between possible asthma on the one hand and rhinitis, furred pet, pollen and food allergy tended to increase by age, while possible asthma-eczema co-occurrence appeared to be more stable across age.

A corresponding analysis (Table 1, mid-section) showed that presence of possible asthma, eczema, food, and pollen allergy were all highly significantly related to presence of rhinitis. Pollen allergy was the far most important determinant, with a Wald’s chi-square value more than four times that of all other variables combined. All co-occurrence by age tended to increase except for eczema, Figure 1b.

For eczema presence (Table 1, right hand section), the most important determinant was food allergy, Wald’s chi-square measure being larger than that of all other variables combined. The co-occurrence increased by age for all variables, with the largest increase at age 2 and age 6, Figure 1c.

## Discussion

In this nationwide study there was considerable co-occurrence between prevalent possible asthma, rhinitis, eczema and food allergy. The prevalence of all conditions peaked at the age of 3 with the exception of rhinitis, where the prevalence increased throughout the whole age span 1–6. Eczema was by far the most common of the conditions. The mean prevalence across age of the other manifestations was approximately the same.

The strengths of the study include that it was based on a large sample of pre-school children covering all of Sweden. As shown in the Settings section, 74% of all Swedish preschool children, and more than 80% of children 3 years or older attend day-care centres. Children in Swedish day care centres may thereby be regarded as representative of all Swedish preschool children. One possible source of bias might have been that children attending DCs were more or less healthy than non-attending children. However, some studies have found that children attending DCs had more respiratory symptoms than children cared for at home [22], while others found no difference [23], and still others have found differences only in specific age groups [24]. At any rate the differences found were small.

Moreover, the parental response rate to the postal questionnaire was 68-71%, which is higher than the approximately 50%- 60% response rate usually obtained in random population samples. The data obtained in this study are therefore equal to or more complete than corresponding data obtained from random population samples. The possible bias caused by non-response may be estimated based on the assumptions that non-respondents, for instance, had a possible asthma on average either five standard error units more often or five standard error units less often then the respondents, i.e., a considerable and highly significant difference. The overall possible asthma prevalence in respondents and non-respondents combined would then have been 9.0% if non-respondents had higher prevalence than respondents, and 8.8% if they had lower prevalence, as compared with the 8.9% we found among respondents. The potential bias owing to non-response is therefore small.

The limitations of the study include that postal questionnaire data was the only possible source of information in a study population of this size. It would have been desirable to have access to medical investigation data, however impractical with a study population of this size and dispersion across the country. The questionnaire used in this study is based on the ISAAC questionnaire, validated and used worldwide. All sources of information have inherent information bias to some extent. Medical history data usually have a low degree of such bias, and according to the validations made it is acceptable for clinical epidemiologic studies, like the present one.

The definitions of possible asthma and allergic manifestations were based on combinations of variables. Possible asthma, for instance, is in most prevalence studies defined only by wheezing during the last year. In a previous publication we were able to show that the prevalence of possible asthma criteria used in the present study was approximately the same per age and sex group, except for wheezing alone during the last year, where the prevalence was almost three times higher (approximately 35% among one-year-old boys and 19% among one-year old girls) [17]. To improve precision and to arrive at a definition as similar as possible to that used in a clinical setting, the criteria used in this study were adopted.

In this study the total prevalence of possible asthma, rhinitis, eczema or food allergy across the age span 1–6 years was 35.7%. A previous Swedish study found a similar total prevalence, 32% [9]. The 8.9% possible asthma prevalence is similar to what others have found [7,9,25]. We found a total rhinitis prevalence of 8.1%. Other western and northern European studies have reported similar prevalence levels [3,9]. The eczema prevalence found in this study, 21.7%, is similar to results from other Swedish studies [3,26], but higher than results reported from other European and non-European countries in the ISAAC study [3]. The food allergy prevalence in the present study was 6.6%. Other Scandinavian and British studies reported 8%-15%, based on questionnaires [4,27,28], but only 2%-3.5% after oral provocation [5,27-30]. The results from this study are thus similar to those from other Scandinavian studies, but they differ to some extent from data collected in other parts of the world.

We found a highly significant co-occurrence between the various atopic manifestations. Pet allergy and rhinitis had the largest impact effect of the allergy manifestations on the presence of possible asthma. Regarding rhinitis, pollen allergy had by far the largest impact. For eczema, food allergy had the unquestionably largest impact. These findings are well in line with clinical observations. Results from other studies are usually based on two-by-two manifestation comparisons, with a few exceptions [10-12,30,31] and with only partial adjustments, which means that the extent of the co-occurrence is only partially shown.

There are two interesting issues linked to co-occurrence: cause or causes and consequences. It is unlikely that the various atopic manifestations cause or trigger each other, it is much more likely that there are common underlying factors that trigger the onset of manifestations. These are presently incompletely known. It is a commonly held view that the manifestations become prevalent in some sort of ordered sequence, such as the ‘atopic march’ [12-14]. So far, no preventive action may be based on these circumstances.

However, in a German birth cohort study, children with moderate to severe early eczema often had early wheeze. Of these children, half had wheeze before the onset of eczema and half had eczema before or concomitant with wheezing [14]. As early as at 2 years of age, 6.5% of the children in a Swedish birth cohort study had at least three of five atopic manifestations [32]. In the present study co-occurrences were common as early as before 3 years of age. The impact of pollen allergy as compared with that of furred pet allergy on rhinitis was larger than expected in these young children and is particularly noteworthy.

## Conclusions

In conclusion, all but one manifestation had peak prevalence at 3 years of age. Although the present study is based on cross-sectional data, where the manifestations among individual children were not followed over time, there was no indication that the manifestations appeared in rank order. They rather appeared to have their onset at about the same time, possibly with the exception of rhinitis. Co-occurrence between parentally reported possible asthma and the atopic manifestations was common, and some combinations were more common than others.

## Consent

Written informed consent was obtained from the parents for the publication of this report and any accompanying images.

## Competing interests

The authors declare that they have no competing interests.

## Authors’ contributions

All authors participated in the design of the study. KB and KS performed the sampling of the study population. KB, ME and KS performed the monitoring of the data collection and corrected and revised the data files. KB and KS performed the analyses and drafted the manuscript. All authors participated in the discussions and revisions of the manuscript, and all authors have seen and approved the final version.

## Pre-publication history

The pre-publication history for this paper can be accessed here:

http://www.biomedcentral.com/1471-2458/13/764/prepub

## Supplementary Material

Additional file 1 Supplementary questions. Click here for file
