# Supplementary material for: Prevalence and co-occurrence of parentally reported possible asthma and allergic manifestations in pre-school children
Source: BMC Public Health. 2013 Aug 16;13:764. doi: 10.1186/1471-2458-13-764 (PMC3765705; doi:10.1186/1471-2458-13-764)
Supplement: Additional file 1 — Supplementary questions. [file 1471-2458-13-764-S1.doc]

| **Supplementary questions** | **Response alternatives** |
| --- | --- |
| Has your child had an asthma diagnosis by a physician? | Yes  No |
| Has your child had a hay fever or an allergic rhinitis diagnosis by a physician? | Yes  No |
| Has your child had an eczema diagnosis by a physician? | Yes  No |
| Have you noticed that your child is sensitive to cats? | Yes  No |
| If no, have you suspected that your child is sensitive to cats? | Yes  No |
| Have you noticed that your child is sensitive to dogs? | Yes  No |
| If no, have you suspected that your child is sensitive to dogs? | Yes  No |
| Have you noticed that your child is sensitive to horses? | Yes  No |
| If no, have you suspected that your child is sensitive to horses? | Yes  No |
| Have you noticed that your child is sensitive to rodents? Guinea pig, rabbit, hamster, rat etc. | Yes  No |
| If no, have you suspected that your child is sensitive to rodents? Guinea pig, rabbit, hamster, rat etc. | Yes  No |
| Have you noticed that your child is sensitive to pollen? | Yes  No |
| If no, have you suspected that your child is sensitive to pollen? | Yes  No |
| Report all drugs that your child has used during the last 12 months, for example tablets, ointments, nasal-drops, eye-drops, inhalation spray, inhalation powder. Indicate the name of the drug, the strength and dose (that is how much the child takes every time and how often.) | for example 2 tablets 3 times daily |
| Has your child a food allergy? | Yes  No |
| If yes, which kind of food is your child allergic to?  Milk  Egg  Fish  Peanuts  Nuts  Soy  Stone fruits (apples, pears, cherries, plums, peaches, nectarines) | Yes or No to each food item |
| If other food allergy, report each kind of food. | Free text |
| Which education has the father of the child? | Nine-year compulsory education  Two-year upper secondary education  Three-four-year upper secondary education  University or college education |
| Which education has the mother of the child? | Nine-year compulsory education  Two-year upper secondary education  Three-four-year upper secondary education  University or collegeeducation |
| Is anybody smoking indoor in the child’s home? | Yes, daily  Yes, often 1-4 times a week  Yes, sometimes 1-3 times a month  No, never |
| Did anybody smoke at home during the child’s first year of life? | The father was smoking  The mother was smoking  Another member of the family was smoking |
| Was the child’s mother a smoker during the pregnancy? | Yes  No |
